# Variable Gain Robust Estimation Algorithm Based on Quality Evaluation of Measurement Information

**DOI:** 10.3390/s26206368

**Published:** 2026-10-09

**Authors:** Chunlei Gao, Bin Zhao, Ying Zhu

**Affiliations:** 1 Jincheng College, Nanjing University of Aeronautics and Astronautics, Nanjing 211156, China; 2 School of Marine and Electrical Engineering, Jiangsu Maritime Institute, Nanjing 211170, China; zhaobin@nuaa.edu.cn; 3 School of Electrical and Power Engineering, Hohai University, Nanjing 211100, China; yingzhu@hhu.edu.cn

**Keywords:** integrated navigation, smooth bounded layer, measurement quality evaluation, variable gain estimation

## Abstract

Given that satellite signals are susceptible to interference, which causes performance degradation of Kalman estimation, and that the smooth variable structure estimation algorithm has limited applications in integrated navigation, this paper proposes a variable gain robust estimation algorithm based on the quality evaluation of measurements. A measurement quality evaluation index based on the smooth bounded layer is constructed. When the measurements are normal, the Kalman estimation gain is adopted to obtain the optimal estimation. When the measurements are abnormal, the smooth variable structure estimation and the robust adaptive estimation algorithm are combined: for the state quantities corresponding to the measurements, the smooth variable structure estimation gain is used to resist measurement abnormalities; for the state quantities without corresponding measurements, the measurement information evaluation index is used to reconstruct the filtering gain, so as to reduce the impact of abnormal data on the fusion results. The verification results show that under various typical GNSS faults, the proposed algorithm can maintain good estimation accuracy, and can quickly converge to the accuracy under normal conditions after the fault ends, thus improving the robustness of the airborne SINS/GNSS integrated navigation system. Based on the Monte Carlo experimental results, compared with the Kalman filter, the smooth variable structure filter and the innovation-based adaptive Kalman filter, the platform misalignment angle accuracy of the proposed variable gain robust estimation algorithm is improved by more than 10%, the velocity accuracy by more than 26%, and the position accuracy by more than 56%.

## 1. Introduction

During flight missions, unmanned aerial vehicles (UAVs) often encounter a variety of complex and harsh environments, which in turn have a significant impact on the operational reliability of various navigation systems [1,2,3]. In view of this, it is necessary to fully explore and utilize the information resources of airborne navigation systems to achieve complementary advantages among different systems. Furthermore, in-depth research on robust filtering algorithms with wide applicability should be carried out to provide technical support for the effective fusion of navigation information [4]. Based on the complementary characteristics of the Strapdown Inertial Navigation System (SINS) and the Global Navigation Satellite System (GNSS), the SINS/GNSS integrated navigation technology has occupied a dominant position in the field of airborne integrated navigation [5].

To address the issue of degraded Kalman estimation performance caused by system modeling biases and noise uncertainties, the Habibi team developed a model-based smooth variable structure filtering (SVSF) method [6]. This algorithm employs a discontinuous gain mechanism with a variable structure, enabling state estimates to converge within the true value interval. It exhibits excellent robust performance against bounded model and noise uncertainties.

The initial application scenario of the smooth variable structure filtering algorithm was the electro-hydraulic actuator drive system, where all state variables in the system had a direct corresponding relationship with the measured values [7]. Subsequently, the application scope of this algorithm was expanded to the field of target tracking, where the measured values are directly related to the position state variables [8], and it has demonstrated good accuracy and robustness in the above-mentioned fields. A modified smooth variable structure filter with an optimally variable bounded layer is proposed. This filter is applied to electro-hydraulic actuators, verifying its strong robustness against modeling errors [9]. The interactive multi-model idea is combined with the characteristics of the smooth variable structure filter. The robustness and stability of the proposed algorithm are verified in maneuvering aircraft target tracking under uncertain motion models and observation information [10]. To overcome the limitation of the Kalman filter on the Gaussian characteristics of noise, a combined filtering algorithm of smooth variable structure and Kalman filter is designed, which effectively improves target positioning accuracy and algorithm robustness under strong disturbances and uncertain noise [11]. A novel lattice smooth variable structure filter (LSVSF) designed for maneuvering target tracking under model uncertainty is proposed. Simulation results show that the proposed LSVSF algorithm achieves superior tracking accuracy and higher robustness compared to conventional methods [12]. A distributed cubature smooth variable structure fusion algorithm based on matrix weighting is proposed, which effectively overcomes the influence of model uncertainty and cross-correlated noise and has higher state estimation accuracy [13]. The SVSF filtering strategy is incorporated into the PHD filtering framework [14]. Experimental results under complex tracking scenarios indicate that the combined-strategy filters remained stable, whereas the standard PHD filters diverged due to the scenario’s complexity. A novel state estimation method based on the Mixture Generalized Minimum Error Entropy smooth variable structure filter (MGMEE-SVSF) is presented. The proposed algorithm achieves better robustness and smaller tracking errors compared with existing approaches [15].

However, the conventional smooth variable structure filter is based on the premise that state variables and measurements correspond one-to-one [16], and it only corrects the state variables directly corresponding to the measurements. In the SINS/GNSS integrated navigation system, the state vector includes the carrier’s attitude, velocity, position, and inertial device errors. The dimension of measurement information is generally much smaller than that of state variables, so the advantages of the smooth variable structure filter have not been fully utilized. Moreover, providing directly corresponding measurements for all state variables is not only costly but also unnecessary in practical applications.

Based on the analysis of the parameter indicators and algorithm characteristics of integrated navigation information fusion algorithms, this paper proposes a variable gain robust estimation algorithm based on measurement quality evaluation. The main novelties of this work are summarized as follows:

(1) A measurement quality evaluation index is constructed based on the width of the smooth bounded layer, which incorporates both prior and posterior measurement errors and improves the detection sensitivity for incipient gradual faults and small-amplitude step faults.
(2) A dimension-wise gain reconstruction mechanism is designed for the abnormal measurement mode. When measurements are abnormal, an equivalent weight matrix is built to reconstruct filtering gains, enabling independent adjustment of each gain component rather than global uniform scaling for the whole measurement vector.
(3) A state partitioning strategy is developed for the high-dimensional state vector of SINS/GNSS integrated navigation. System states are divided into measurable states (velocity and position with direct GNSS observations) and non-measurable states (platform misalignment angles and inertial sensor errors without corresponding measurements), and different gain update rules are adopted for the two categories of states.
(4) A threshold-driven mode switching logic is established to govern the switch between the normal measurement KF mode and the abnormal measurement composite SVSF adaptive robust estimation mode according to the output of the measurement quality evaluation index. Meanwhile, saturation interpolation within the smooth bounded layer suppresses abrupt state jumps at switching instants.

This method achieves comprehensive performance improvement for UAV integrated navigation by fully combining the high-precision property of Kalman estimation, the robustness advantage of smooth variable structure estimation, and the fault-tolerant capability of adaptive estimation.

## 2. Model of SINS/GNSS Integrated Navigation System

### 2.1. State Equation of SINS/GNSS Integrated Navigation System

The state variables of the integrated navigation filter are selected as follows: the mathematical platform misalignment angle of the strapdown inertial navigation system $\phi_{E},\phi_{N},\phi_{U}$, velocity error $\delta v_{E},\delta v_{N},\delta v_{U}$, position error δL,δλ,δh, random constant drift of the gyroscope $\varepsilon_{x},\varepsilon_{y},\varepsilon_{z}$, and zero bias of the accelerometer $\nabla_{x},\nabla_{y},\nabla_{z}$.

$$X(t)={\left[\phi_{E}\;\;\;\phi_{N}\;\;\;\phi_{U}\;\;\;\delta v_{E}\;\;\;\delta v_{N}\;\;\;\delta v_{U}\;\;\;\delta L\;\;\;\delta \lambda \;\;\;\delta h\;\;\;\varepsilon_{x}\;\;\;\varepsilon_{y}\;\;\;\varepsilon_{z}\;\;\;\nabla_{x}\;\;\;\nabla_{y}\;\;\;\nabla_{z}\;\;\;\right]}^{T}\;\;\;$$

Through the analysis of the performance and error sources of the SINS system, the state equation of the integrated navigation system can be obtained:

(1) $$\dot{X}(t)=\Phi (t)X(t)+\Gamma (t)W(t)$$

where X(t) is the system state vector; Φ(t) is the state transition matrix; Γ(t) is the system noise coefficient matrix; W(t) is the system noise vector.

### 2.2. Measurement Equation of SINS/GNSS Integrated Navigation System

Taking the differences between the position and velocity obtained from the navigation solution of the strapdown inertial navigation system and those measured by the satellite navigation system as the measurement, the measurement equation of the SINS/GNSS integrated navigation filter is thus derived:

(2) $$\begin{array}{l} Z(t)=H(t)X(t)+V(t)\\ \;\;\;\;\;\;\;\;=\left[\begin{array}{c} Z_{v}(t)\\ Z_{p}(t) \end{array}\right]=\left[\begin{array}{c} H_{v}(t)\\ H_{p}(t) \end{array}\right]X(t)+\left[\begin{array}{c} V_{v}(t)\\ V_{p}(t) \end{array}\right]\\ \;\;\;\;\;\;\;\;=\left[\begin{array}{ccc} 0_{6\times 3} & I_{6\times 6} & 0_{6\times 6} \end{array}\right]X(t)+V(t) \end{array}$$

where Z(t) is the measurement vector; H(t) is the measurement coefficient matrix; V(t) is the measurement noise vector; $Z_{v}(t)$ is the velocity measurement vector; $H_{v}(t)$ is the velocity measurement coefficient matrix; $V_{v}(t)$ is the velocity measurement noise vector, approximately white noise; $Z_{p}(t)$ is the position measurement vector; $H_{p}(t)$ is the position measurement coefficient matrix; $V_{p}(t)$ is the position measurement noise vector, approximately white noise; $I_{6\times 6}$ is the identity matrix.

By discretizing the state equation and measurement equation, the discrete system model shown as follows is obtained:

(3) $$\left\{\begin{array}{l} X_{k}=\Phi_{k,k-1}X_{k-1}+\Gamma_{k-1}W_{k-1}\\ Z_{k}=H_{k}X_{k}+V_{k} \end{array}\right.$$

Based on Equation (3), an information fusion algorithm can be applied for filtering estimation to achieve airborne SINS/GNSS integrated navigation.

## 3. Information Fusion Algorithm and Analysis

### 3.1. Kalman Filtering Algorithm

Given that the Kalman filtering algorithm is quite mature and has been widely applied, this section directly presents its filtering recurrence equations as follows [17]:

(4) $$\left\{\begin{array}{c} {\hat{X}}_{k/k-1}=\Phi_{k/k-1}{\hat{X}}_{k-1}\\ P_{k/k-1}=\Phi_{k/k-1}P_{k-1}\Phi_{k/k-1}^{T}+\Gamma_{k-1}Q_{k-1}\Gamma_{k-1}^{T}\\ K_{k}=P_{k/k-1}H_{k}^{T}{\left[H_{k}P_{k/k-1}H_{k}^{T}+R_{k}\right]}^{-1}\\ {\hat{X}}_{k}={\hat{X}}_{k/k-1}+K_{k}[Z_{k}-H_{k}{\hat{X}}_{k/k-1}]\\ P_{k}=[I-K_{k}H_{k}]P_{k/k-1} \end{array}\right.$$

where ${\hat{X}}_{k/k-1}$ is the one-step state prediction; $P_{k/k-1}$ is the one-step predicted state error covariance matrix; $P_{k}$ is the posterior state error covariance matrix; $K_{k}$ is the Kalman gain; $Q_{k-1}$ and $R_{k}$ represents the process and measurement noise covariance matrices, respectively; I represents the identity matrix.

When the model and noise characteristics of the integrated navigation system are both accurately known, optimal state estimation of the integrated navigation can be obtained by means of the Kalman filtering equation described in Equation (4). This provides a clear mathematical expression and implementation approach for the theoretical optimal solution of integrated navigation.

However, in practical application scenarios, the above ideal conditions are often difficult to achieve. When uncertainties exist in the model of the integrated navigation system or the noise characteristics cannot be accurately known, the performance of the Kalman filter will degrade significantly. This is because the Kalman filtering algorithm strongly depends on the system model and noise statistical characteristics. Model deviations or non-ideal noise characteristics will interfere with the filtering process, thereby reducing estimation accuracy and deteriorating the filter stability [18,19].

### 3.2. Smooth Variable Structure Filtering Algorithm

**Figure 1:**
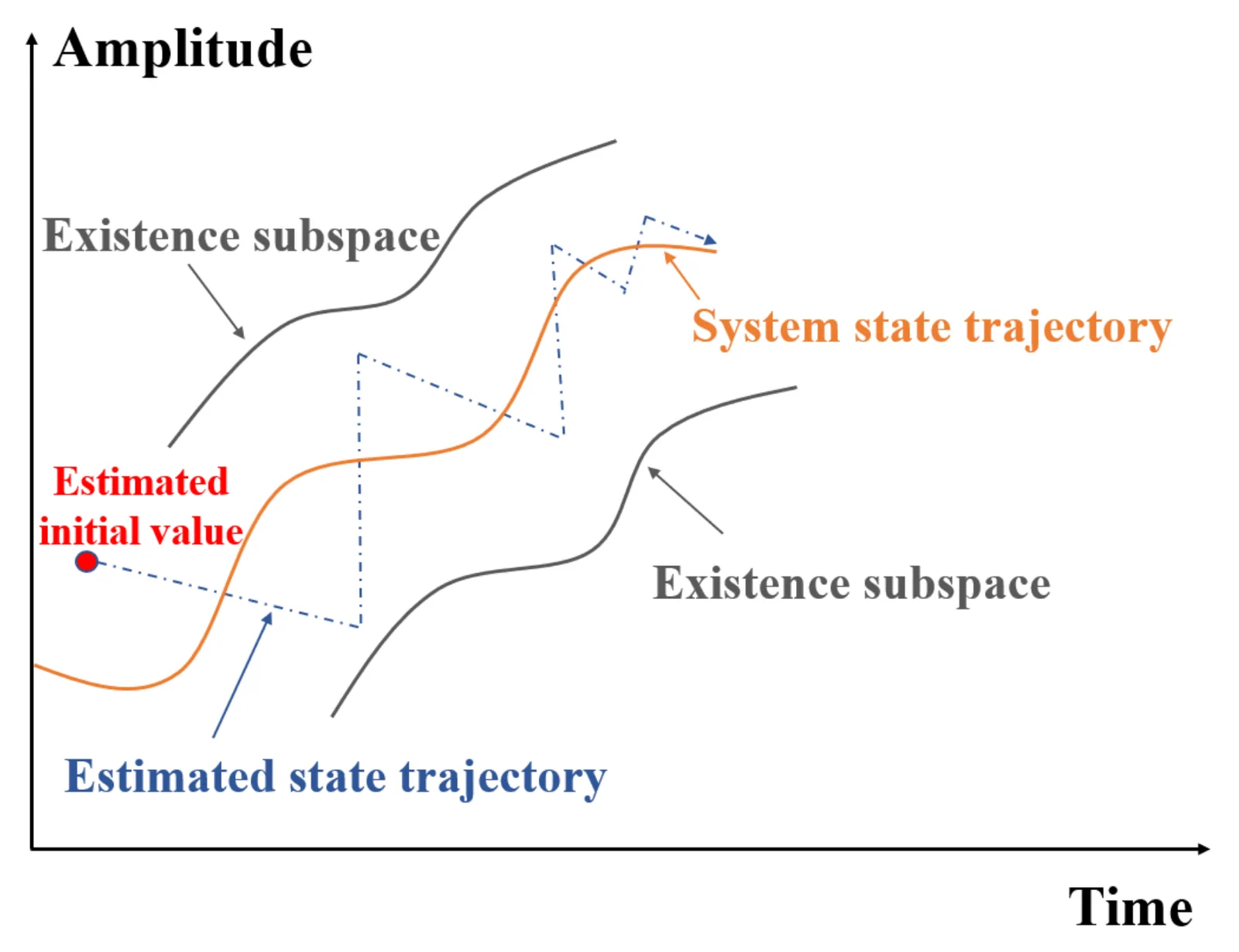
Basic principle of SVSF estimation.

As shown in Figure 1, the system state trajectory evolves over time. Based on the smooth variable structure theory, the estimated state is generated based on the system’s uncertain model. By selecting an appropriate smooth variable structure gain, the estimated state trajectory can gradually approach the system state trajectory until it enters the existence subspace. After that, the estimated state is confined within this subspace and oscillates around the true state trajectory [20]. The existence subspace always surrounds the true state trajectory of the system, and its width covers system disturbances and their uncertainties. If the disturbances and uncertainties are bounded, the width of the existence subspace can be determined. Therefore, the SVSF algorithm can still maintain good stability and robustness when there is a mismatch between the system model and the noise statistics.

The concept of smooth bounded layer width is introduced into the SVSF algorithm. This width affects the average estimation accuracy and essentially reflects the interference level imposed on the filter. The specific calculation formula is as follows.

(5) $$\psi_{k}={\left[{\left[\mathrm{diag}\left(\left|e_{z,k/k-1}\right|+\gamma \left|e_{z,k-1/k-1}\right|\right)\right]}^{-1}H_{k}P_{k/k-1}H_{k}^{T}S_{k}^{\;-1}\right]}^{-1}$$

where $\psi_{k}$ is the smooth bounded layer width; $\mathrm{diag}\left(\cdot \right)$ represents the diagonal matrix operator; $\gamma \left(0<\gamma <1\right)$ is the convergence factor; $e_{z,k-1/k-1}$ and $e_{z,k/k-1}$ are the posterior measurement residual and the prior measurement innovation respectively:

(6) $$\left\{\begin{array}{c} e_{z,k-1/k-1}=Z_{k-1}-H_{k-1}{\hat{X}}_{k-1}\\ e_{z,k/k-1}=Z_{k}-H_{k}{\hat{X}}_{k/k-1} \end{array}\right.$$

where $S_{k}$ is the measurement error covariance matrix

(7) $$\begin{array}{l} S_{k}=E\left[e_{z,k/k-1}e_{z,k/k-1}^{T}\right]=E\left[\left(Z_{k}-H_{k}{\hat{X}}_{k/k-1}\right){\left(Z_{k}-H_{k}{\hat{X}}_{k/k-1}\right)}^{T}\right]\\ \;\;\;\;=H_{k}P_{k/k-1}H_{k}^{T}+R_{k} \end{array}$$

and where $E\left[\cdot \right]$ denotes the expectation operator.

With the smooth bounded layer calculated, the SVSF gain $K_{k}^{SVSF}$ can be obtained as follows:

(8) $$K_{k}^{SVSF}=H_{k}^{+}\mathrm{diag}\left[\left(\left|e_{z,k/k-1}\right|+\gamma \left|e_{z,k-1/k-1}\right|\right)\circ \mathrm{sat}\left(\frac{e_{z,k/k-1}}{\psi_{k}}\right)\right]{\left[\mathrm{diag}\left(e_{z,k/k-1}\right)\right]}^{-1}$$

where the superscript “+” denotes the matrix pseudoinverse operation; “∘” denotes the Hadamard product operation; $\mathrm{sat}\left(\frac{e_{z,k/k-1}}{\psi_{k}}\right)$ is the saturation function, which is defined as follows:

(9) $$\mathrm{sat}\left(\frac{e_{z,k/k-1}}{\psi_{k}}\right)=\left\{\begin{array}{c} 1,\frac{e_{zi,k/k-1}}{\psi_{kii}}\geq 1\\ \frac{e_{zi,k/k-1}}{\psi_{kii}},-1<\frac{e_{zi,k/k-1}}{\psi_{kii}}<1\\ -1,\frac{e_{zi,k/k-1}}{\psi_{kii}}\leq -1 \end{array}\right.\left(i=1,2,\ldots ,m\right)$$

where $e_{zi,k/k-1}$ is the *i*-th element of $e_{z,k/k-1}$, and $\psi_{kii}$ is the *i*-th diagonal element of $\psi_{k}$.

Through the above saturation function, robustness and stability can be ensured outside the smooth bounded layer; inside the smooth bounded layer, the filtering gain is interpolated to form a smooth function, thereby mitigating the chattering phenomenon.

According to the SINS/GNSS integrated navigation system model constructed above, the state vector is 15-dimensional, including navigation parameters and inertial device parameters, while the measurement vector only consists of 6-dimensional position and velocity information from the satellite navigation system, so its dimension is lower than that of the state vector. The corresponding measurement coefficient matrix is $H_{k}=\;\;\;\left[\begin{array}{ccc} 0_{6\times 3} & I_{6\times 6} & 0_{6\times 6} \end{array}\right]$; therefore, $H_{k}^{+}={\left[\begin{array}{ccc} 0_{3\times 6} & I_{6\times 6} & 0_{6\times 6} \end{array}\right]}^{T}$, and substituting it into Equation (8) yields the smooth variable structure gain:

(10) $$K_{k}^{SVSF}≜\left[\begin{array}{c} 0_{3\times 6}\\ {\left(\times \right)}_{6\times 6}\\ 0_{6\times 6} \end{array}\right]$$

where ${\left(\times \right)}_{6\times 6}$ denotes the module of non-zero elements.

It can be seen that in the SINS/GNSS integrated navigation system, the dimension of the measurement vector is much smaller than that of the state vector. Among the 15-dimensional state variables, only velocity and position correspond directly to the measurements, while the platform misalignment angles and inertial device errors have no corresponding measurements. Consequently, the rows of the SVSF gain corresponding to these unmeasurable states become all-zero, and then according to the posterior state estimation equation:

(11) $${\hat{X}}_{k}={\hat{X}}_{k/k-1}+K_{k}^{SVSF}\left(Z_{k}-H_{k}{\hat{X}}_{k/k-1}\right)≜{\hat{X}}_{k/k-1}+\left[\begin{array}{c} 0_{3\times 6}\\ {\left(\times \right)}_{6\times 6}\\ 0_{6\times 6} \end{array}\right]\left(Z_{k}-H_{k}{\hat{X}}_{k/k-1}\right)$$

It can be seen that only the velocity and position state estimates that directly correspond to the measurement are corrected by the measurement innovation ($Z_{k}-H_{k}{\hat{X}}_{k/k-1}$), while the platform misalignment angle and inertial device errors cannot be effectively corrected via the measurement innovation. This leads to error accumulation and mutual coupling, degrading the performance of integrated navigation. As a result, the robustness of the SVSF algorithm cannot be fully exploited in the SINS/GNSS integrated navigation system.

## 4. Variable Gain Robust Estimation Algorithm Based on Measurement Quality Evaluation

### 4.1. Principle of Variable Gain Robust Estimation

Based on the previous analysis, it can be concluded that: when the system model and noise characteristics are accurately known, the Kalman estimation can provide the optimal state estimation for integrated navigation; the smooth variable structure estimation, through its unique parameter adjustment mechanism, can effectively resist various interferences and ensure the stability and reliability of the filtering process. However, because the dimension of the measurements in the SINS/GNSS integrated navigation system is much smaller than that of the state vector, the advantages of the SVSF algorithm fail to be fully exerted.

In addition, it can be observed from Equation (5) that the smooth bounded layer width, as a function, contains both prior and posterior measurement error variables, directly reflecting the quality of the measurements. Based on this, this paper adopts the smooth bounded layer width to construct the measurement quality evaluation index for SINS/GNSS integrated navigation.

During the filtering operation, the smooth bounded layer width corresponding to the measurement of each dimension is detected in real time, which is used to construct the measurement quality evaluation function:

(12) $$\lambda_{ki}=\left(\psi_{kii}-{\bar{\psi }}_{i}\right)/\sigma_{i}$$

where $\lambda_{ki}$ is the measurement quality evaluation statistic for the *i*-th dimension at time k; $\psi_{kii}$ denotes the width of the smooth bounded layer for the *i*-th dimension, i.e., the *i*-th diagonal element of the smooth bounded layer width matrix $\psi_{k}$; ${\bar{\psi }}_{i}$ is the mean value of the smooth bounded layer width of the i-th dimension; and $\sigma_{i}$ is the root mean square error of the smooth bounded layer width of the i-th dimension.

By comparing $\lambda_{ki}$ with the detection threshold c, the validity of the i-th dimension measurement can be judged in real time. $\lambda_{ki}$ denotes the standardized measurement quality evaluation statistic, which approximately follows the standard normal distribution under fault-free conditions. For normal measurement information, $\left|\lambda_{ki}\right|$ < 2.5 corresponds to a confidence level of 98.76%, while $\left|\lambda_{ki}\right|$ < 3 corresponds to a confidence level of 99.73%. Therefore, the threshold range c=2.5~3 is generally adopted as the judgment threshold for measurement faults [21].

A sensitivity analysis of threshold c is discussed as follows. Increasing the threshold suppresses false alarms, but weakens the capability to detect small faults, resulting in a lower fault detection rate, higher missed detection rate and longer detection delay. Reducing the threshold can capture weak faults more easily while introducing more false alarms.

To further quantitatively illustrate the influence of threshold selection on detection performance, additional tests under different thresholds ($c=\begin{array}{cccc} 2.0, & 2.5, & 3.0, & 3.5 \end{array}$) are carried out, adopting exactly the same simulation configuration and fault injection scenarios as Section 5.1. The fault detection rate, missed detection rate, false alarm rate and detection delay under different thresholds are summarized in Table 1. Detailed simulation parameters, fault scenarios and detection curves are presented in Section 5.1 and will not be repeated here.

It can be observed from Table 1 that the experimental results are consistent with the above theoretical qualitative analysis. As threshold c increases, the false alarm rate continuously decreases, whereas the missed detection rate and average detection delay gradually rise. c=2 and c=2.5 yield high fault detection rates, but suffer from relatively high false alarm rates. When c rises from 3.0 to 3.5, no further performance improvement can be observed. Considering the comprehensive trade-off among fault detection capability, false alarm suppression and detection timeliness, c=3 is finally selected as the detection threshold for all subsequent simulation tests in this paper.

Based on the evaluation results of measurement quality, a variable gain integrated navigation information fusion method for the smooth bounded layer is proposed:

when $\lambda_{ki}\leq c$, the measurement is normal, and the Kalman estimation gain given in Equation (4) is used for information fusion to obtain the optimal state estimation of integrated navigation;

when $\lambda_{ki}>c$, the measurement is abnormal. The smooth variable structure estimation and robust adaptive estimation are combined to comprehensively ensure the accuracy and robustness of the state estimation.

For state variables directly corresponding to measurements, the SVSF gain given in Equation (8) is adopted to resist measurement anomalies; for state variables without corresponding measurements, the measurement quality evaluation index parameters are used to reconstruct and robustly tune the Kalman estimation gain in real time, so as to weaken the adverse impact of abnormal data on the fusion results.

(13) $$K_{k}^{AKF}=P_{k/k-1}H_{k}^{T}{\left(H_{k}P_{k/k-1}H_{k}^{T}+\alpha_{k}^{-1}R_{k}\right)}^{-1}$$

where $\alpha_{k}=\mathrm{diag}\left[\alpha_{k1},\alpha_{k2},\ldots ,\alpha_{kn}\right]$ is the equivalent weight matrix constructed based on the smooth bounded layer, and each robust equivalent weight element is calculated as follows:

(14) $$\alpha_{ki}=\min\left\{\begin{array}{cc} 1, & \frac{c}{\lambda_{ki}} \end{array}\right\}$$

In the SINS/GNSS integrated navigation system, when $\lambda_{ki}>c$, by combining $K_{k}^{SVSF}$ and $K_{k}^{AKF}$, we can obtain:

(15) $$K_{k}=\left[\begin{array}{ccc} K_{k}^{AKF}\left(1:3,:\right); & K_{k}^{SVSF}\left(4:9,:\right); & K_{k}^{AKF}\left(10:15,:\right) \end{array}\right]$$

where $\left(1:3,:\right)$, $\left(4:9,:\right)$, and $\left(10:15,:\right)$ denote the row indices of the state vector for different state components.

Based on Equation (15), under abnormal measurement conditions, the Kalman estimation gain is robustly adjusted via the equivalent weight matrix. According to the correspondence between measurements and state variables, the filtering gain is reconstructed in real time by combining the smooth variable structure estimation gain, thereby suppressing the effect of outliers and enhancing estimation precision.

**Figure 2:**
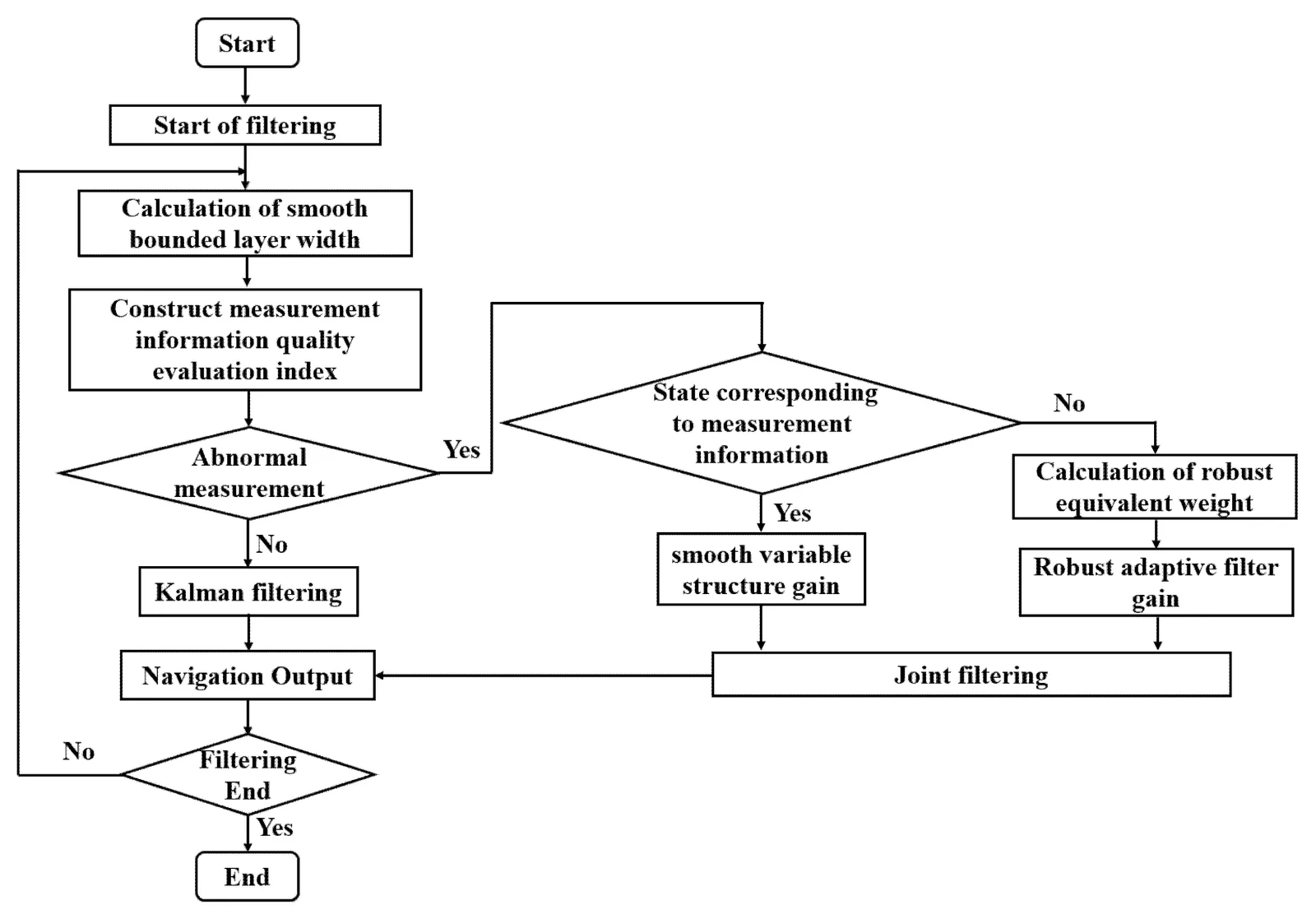
Flowchart of variable gain robust estimation algorithm based on measurement quality evaluation.

To sum up, the flowchart of the variable gain robust estimation algorithm based on measurement quality evaluation is shown in Figure 2.

According to the above-mentioned design scheme, a measurement quality evaluation index is constructed based on the smooth bounded layer to carry out variable gain reconstruction and robust adaptive adjustment for the integrated navigation system. It makes full use of the high-precision property of Kalman estimation, the high robustness advantages of smooth variable structure estimation, and the fault-tolerant capability of adaptive estimation, and comprehensively improves the overall performance of the integrated navigation system through multi-dimensional fusion.

Based on the above algorithm principle analysis, Table 2 provides a theoretical comparison of KF (Kalman filter), SVSF (smooth variable structure filter), AKF (innovation-based adaptive Kalman filter) and the proposed VGAF (variable gain robust estimation algorithm) from four perspectives: measurement quality evaluation index, gain reconstruction, state partitioning strategy and switching logic.

The above theoretical comparison further reveals the intrinsic differences among these filtering frameworks. The performance of these algorithms will be verified by simulation and field experiments in the following sections.

### 4.2. Stability Analysis Based on Switched System Theory

The proposed variable gain robust estimation algorithm is essentially a discrete-time switched system driven by the measurement quality evaluation mechanism. The filter switches between two working subsystems according to the threshold judgment of the index $\lambda_{ki}$: the standard Kalman filter for normal measurement conditions and the composite SVSF and adaptive robust filter for abnormal measurement conditions. To verify the stability of the switching filtering process, this subsection derives the error propagation upper bound and proves the uniform ultimate boundedness (UUB) of the state estimation error triggered by the proposed measurement quality evaluation function.

The discrete state and measurement equations of the SINS/GNSS integrated navigation system are shown in Equation (3). Define the state estimation error as:

(16) $${\tilde{X}}_{k}=X_{k}-{\hat{X}}_{k}$$

where $X_{k}$ denotes the true system state and ${\hat{X}}_{k}$ denotes the filtered state estimation.

For the two switching subsystems, the unified error dynamic equation can be expressed as:

(17) $${\tilde{X}}_{k+1}=\left(\Phi_{k+1,k}-K_{k+1}^{i}H_{k+1}\Phi_{k+1,k}\right){\tilde{X}}_{k}+\left(\Gamma_{k}-K_{k+1}^{i}H_{k+1}\Gamma_{k}\right)W_{k}-K_{k+1}^{i}V_{k+1}$$

where $i\in \left\{1,\left.2\right\}\right.$ is the switching signal determined by the measurement quality evaluation index $\lambda_{ki}$. $K_{k+1}^{1}$ represents the standard Kalman gain under normal measurements, and $K_{k+1}^{2}$ represents the composite robust gain combining SVSF gain and adaptive Kalman gain under abnormal measurements.

Before the boundedness derivation, the necessary and reasonable bounded assumptions for the integrated navigation filtering system are given as follows:

(1) The state transition matrix $\Phi_{k+1,k}$ and observation matrix $H_{k+1}$ are norm-bounded.
(2) The process noise $W_{k}$ and measurement noise $V_{k+1}$ are uniformly bounded, i.e., $\left\|W_{k}\right\|\leq w_{m},\left\|V_{k}\right\|\leq v_{m}$.
(3) The SVSF saturation operation is a bounded mapping, and the adaptive equivalent weight matrix constructed by the smooth bounded layer is strictly bounded.
(4) Both the Kalman gain and the composite robust gain are uniformly bounded matrices during iteration.

These bounded conditions can be justified as follows. The state transition and observation matrices of the SINS/GNSS integrated navigation system are naturally norm-bounded over finite sampling periods. The process and measurement noises are physically bounded owing to the inherent physical constraints of practical navigation sensors. The SVSF saturation function restricts outputs to a finite interval, making the saturation mapping and resultant SVSF gain inherently bounded. The equivalent weight matrix built upon the smooth bounded layer is bounded by the clipping function. For observable systems with positive-definite noise covariances, the Kalman gain satisfies uniform boundedness, a classic result of Kalman filtering. The reconstructed adaptive gain, derived from the bounded equivalent weight matrix, norm-bounded matrices of Assumption (1) and bounded noise in Assumption (2), is also bounded. Since the composite robust gain combines the bounded SVSF gain and reconstructed adaptive gain, it is uniformly bounded.

Taking the 2-norm of the error dynamic equation and applying the triangular inequality for norm estimation:

(18) $$\left\|{\tilde{X}}_{k+1}\right\|\leq \left\|\Phi_{k+1,k}-K_{k+1}^{i}H_{k+1}\Phi_{k+1,k}\right\|\cdot \left\|{\tilde{X}}_{k}\right\|+\left\|\Gamma_{k}-K_{k+1}^{i}H_{k+1}\Gamma_{k}\right\|\cdot \left\|W_{k}\right\|+\left\|K_{k+1}^{i}\right\|\cdot \left\|V_{k+1}\right\|$$

Define the bounded coefficient of the error state transition as $\alpha_{k+1}^{i}=\left\|\Phi_{k+1,k}-K_{k+1}^{i}H_{k+1}\Phi_{k+1,k}\right\|$, and define the bounded noise upper bound as $\beta_{k+1}^{i}=\left\|\Gamma_{k}-K_{k+1}^{i}H_{k+1}\Gamma_{k}\right\|w_{m}+\left\|K_{k+1}^{i}\right\|v_{m}$.

Then the error propagation inequality can be simplified as:

(19) $$\left\|{\tilde{X}}_{k+1}\right\|\leq \alpha_{k+1}^{i}\left\|{\tilde{X}}_{k}\right\|+\beta_{k+1}^{i}$$

Through iterative derivation of the above inequality, the error upper bound in the switching process can be obtained. Under arbitrary switching triggered by the designed measurement quality evaluation function, the state estimation error will stay within a bounded set after finite filtering steps. Consequently, the state estimation error of the proposed variable gain robust filter satisfies uniform ultimate boundedness (UUB). The above derivation theoretically guarantees the stability and boundedness of the entire filtering system under both normal and abnormal measurement conditions.

In addition, although the switching between the two filter subsystems may cause potential state jump discontinuity, the smooth bounded layer with saturation interpolation introduced in the proposed algorithm can effectively suppress abrupt state estimation jumps at switching instants. Combined with the derived uniform ultimate boundedness property, both the global boundedness of estimation error and the suppression of instantaneous switching jumps are guaranteed.

## 5. Experiment and Discussion

### 5.1. Comparative Simulations and Analysis

To verify the performance of the proposed variable gain robust estimation algorithm, a UAV SINS/GNSS integrated navigation platform is constructed for simulation experiments, and the experimental results are analyzed.

The simulation parameters of airborne SINS and GNSS are shown in Table 3.

Step faults and gradual faults are common fault types in satellite navigation systems. During the flight, faults are injected into the GNSS measurements within the time intervals of 300 s–350 s, 500 s–550 s, and 700 s–750 s respectively, as shown in Table 4.

Under the above simulation conditions, four algorithms, namely the proposed variable gain robust estimation algorithm (VGAF), Kalman filter (KF), smooth variable structure filter (SVSF), and a typical innovation-based adaptive Kalman filter (AKF), are compared to verify the performance of the proposed fault detection and variable gain robust estimation algorithm. For the AKF, the chi-square-based Normalized Innovation Squared (NIS) test, a classical innovation-based fault detection scheme for Kalman filters, is adopted as its fault detection module for baseline comparison. AKF constructs the chi-square fault detection function using the innovation of actual and predicted measurements as well as its covariance. Once the function value exceeds the threshold, the measurements are regarded as abnormal, and the Kalman estimation is adaptively tuned according to the detection result.

**Figure 3:**
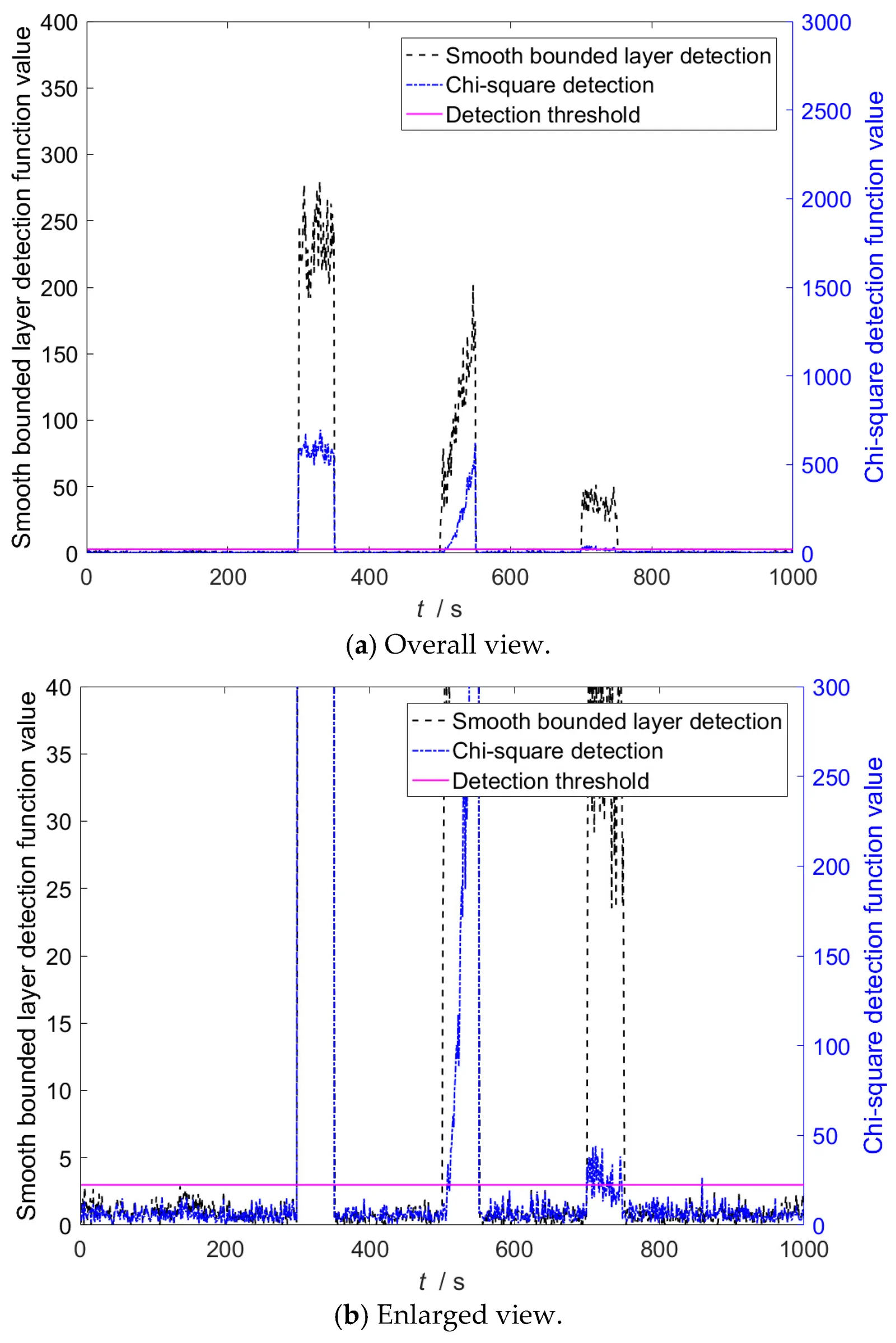
Fault detection function curves of two algorithms. Note: The left vertical axis represents the smooth bounded layer detection statistic, and the right vertical axis denotes the chi-square detection statistic. Although these two indicators have different magnitude scales, their detection threshold lines coincide under the dual-axis scales shown in the figure.

According to the analysis in Section 4.1, the detection threshold for the proposed algorithm is set to 3. The chi-square detection statistic follows the chi-square distribution with six degrees of freedom, and the corresponding threshold is approximately 22.46 at the confidence level of 99.9%. Figure 3 presents the fault detection function curves and enlarged diagrams of VGAF and AKF under the fault scenarios in Table 4.

The statistical indicators such as fault detection rate, missed detection rate, false alarm rate, and detection delay are listed in Table 5.

As shown in Figure 3 and Table 5, for the major step fault within 300 s–350 s, both the traditional chi-square fault detection and the proposed smooth bounded layer fault detection can effectively identify the fault. Both algorithms respond instantaneously upon fault occurrence and show favorable real-time performance. After the fault disappears, the two detection statistics quickly drop below their respective thresholds.

For the gradual change fault in the interval of 500 s–550 s, the chi-square detection statistic crosses the threshold at 507 s, while the detection index based on the smooth bounded layer rises immediately after the gradual change fault appears and triggers an alarm at 501 s.

In the minor step fault period of 700 s–750 s, the chi-square statistic fluctuates near the threshold and suffers from unstable fault identification. By contrast, the proposed smooth bounded layer detection scheme still responds rapidly and steadily exceeds the threshold to capture this minor step fault.

Table 5 further quantifies the detection performance. The smooth bounded layer detection scheme achieves a higher fault detection rate of 98.69%, in contrast to 77.78% for chi-square detection. The missed detection rate of the smooth bounded layer detection is 1.31%, while that of chi-square detection is 22.22%. Meanwhile, both methods maintain very low false alarm rates of 0.35% and 0.23%, respectively. It can be observed that the proposed scheme achieves a significantly higher fault detection rate and a lower missed detection rate, at the cost of a modest increase in the false alarm rate. Nevertheless, its false alarm rate remains at a very low level.

The underlying mechanism is analyzed as follows. The chi-square fault detection statistic is constructed merely using the innovation and its covariance at the current epoch, which only utilizes single-time measurement information. It works satisfactorily for major step faults, but cannot capture minor fault features in the early stage of gradual change faults or minor faults; an alarm is triggered only after the fault accumulates to a sufficiently large amplitude. The smooth bounded layer detection index integrates both the current prior measurement error and the posterior measurement error from the previous epoch, which provides richer information for fault judgment. Compared with the chi-square scheme, the proposed method possesses higher sensitivity and better timeliness for gradual change faults and minor step faults.

**Figure 4:**
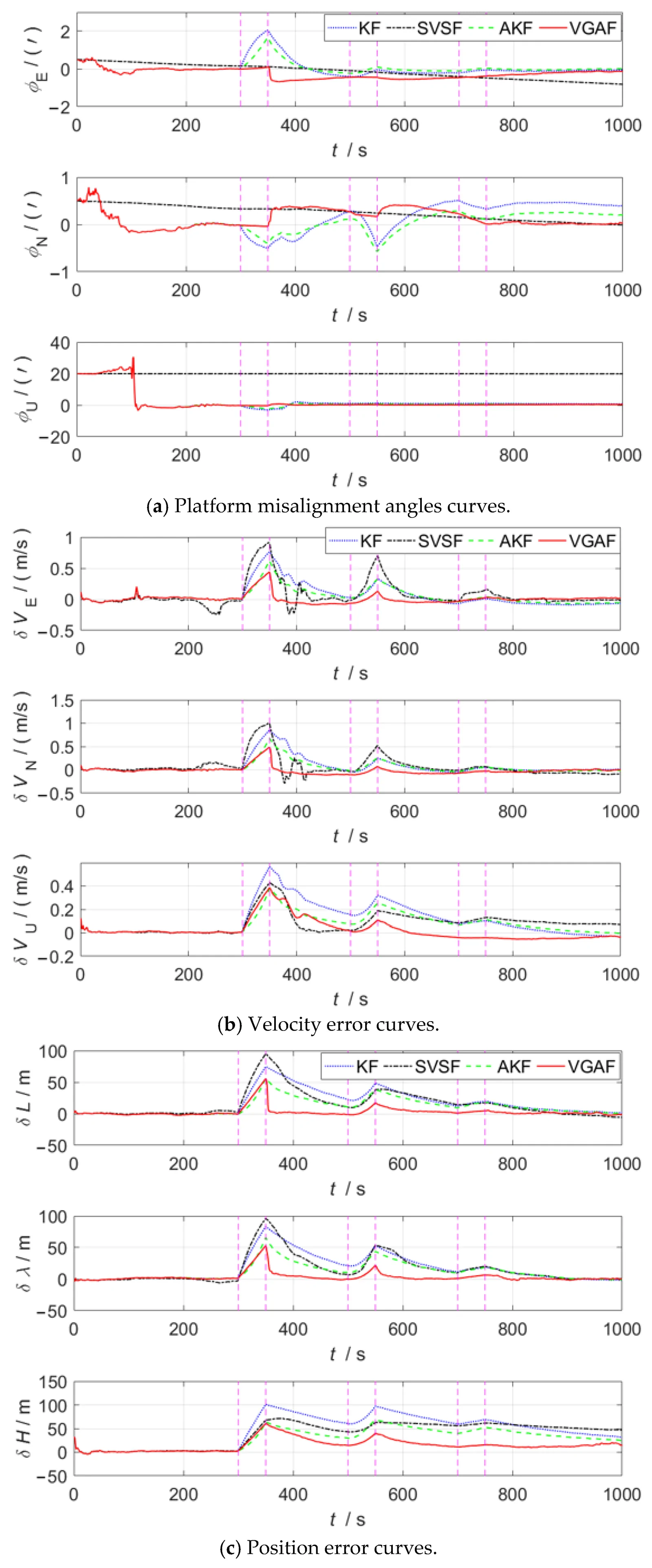
Error curves of the four algorithms.

Figure 4 shows the platform misalignment angle curves, velocity error curves, and position error curves of the four algorithms during integrated navigation.

It can be seen from Figure 4 that the estimation performance of the four algorithms differs significantly under fault-free and various GNSS fault conditions.

(1) In the fault-free period of 0 s–300 s, the proposed VGAF and AKF exhibit comparable accuracy to the conventional KF, both achieving high-precision navigation results. However, SVSF cannot effectively correct the platform misalignment angles due to the lack of corresponding measurements, and its position and velocity are affected by attitude error coupling, which introduces obvious error fluctuations.
  When faults occur in the velocity and position outputs of the satellite navigation system, the measurement noise model of the integrated navigation system becomes mismatched with practical conditions. Under the interference of abnormal measurements, the optimality conditions for the conventional Kalman filter can no longer be satisfied, which leads to degraded state estimation accuracy. During fault duration, navigation errors fluctuate remarkably, system stability deteriorates, and a relatively long convergence period is required after fault clearance.
  The SVSF possesses moderate robustness against bounded disturbances and model uncertainties. Nevertheless, in the SINS/GNSS integrated navigation system, no direct corresponding measurements are available for the platform misalignment angle, so effective correction cannot be obtained through the measurement innovation. Consequently, the platform misalignment angle fails to converge. In the case of satellite navigation system faults, the smooth variable structure estimation suffers from the coupled effects of satellite faults and attitude error coupling, which impairs the robustness of the algorithm, resulting in significant fluctuations in position errors and velocity errors.
(2) During 300 s–350 s, major step faults arise in GNSS velocity and position. Both AKF and the proposed VGAF can detect the fault in real time and perform adaptive adjustment. Comparative results show that VGAF realizes more precise error correction and quicker error convergence with enhanced navigation precision. Since the statistic of chi-square fault detection is only a single scalar, AKF employs this scalar to generate a unified factor for correcting the whole measurement noise covariance matrix. In contrast, VGAF builds an equivalent weight matrix from the detection output of the proposed scheme, which supports independent tuning of measurement noise covariance and filter gain for each dimension and enables dimension-differentiated adjustment.
(3) During 500 s–550 s, gradual change faults are injected into GNSS velocity and position measurements. The chi-square fault detection employed in AKF lacks sufficient sensitivity to gradual change faults, which introduces noticeable detection latency. Navigation errors rise remarkably while the fault persists, and slow error convergence can be observed after fault clearance owing to filter state lag. By contrast, VGAF is capable of rapidly identifying gradual change satellite measurement faults, effectively restraining navigation error oscillations and enhancing the accuracy and stability of navigation results.
(4) From 700 s to 750 s, minor step faults occur in GNSS measurements. Under this scenario, the chi-square detection statistic fluctuates around the threshold and yields unstable fault identification. As a result, AKF cannot reliably trigger adaptive adjustment, leading to filter parameter oscillations. Benefiting from the high sensitivity of the smooth bounded layer detection to minor faults, the proposed VGAF can sense the fault promptly and perform adaptive adjustment. It maintains superior navigation accuracy and output stability under minor step fault conditions.

The proposed VGAF utilizes the width of the smooth bounded layer for GNSS fault detection. When no fault exists, the Kalman gain is adopted for optimal information fusion. Once a satellite fault is detected, VGAF combines smooth variable structure estimation and adaptive estimation: smooth variable structure gain is applied for measurable state variables to resist measurement anomalies, while the Kalman gain is reconstructed and adjusted in real time for unmeasurable state variables using measurement quality evaluation indexes, mitigating the adverse effects of abnormal data. After fault clearance, VGAF can switch back in a timely manner to Kalman estimation and utilize GNSS velocity and position information for rapid correction.

Overall, VGAF achieves balanced performance across major step faults, gradual change faults and small-amplitude step faults. By virtue of the high fault sensitivity of the smooth bounded layer detector and dimension-wise adaptive adjustment via the equivalent weight matrix, VGAF suppresses navigation errors more rapidly under diverse fault modes, converges faster after fault removal, and possesses stronger robustness than KF, SVSF and chi-square-based AKF. This algorithm integrates the high precision of Kalman estimation, the strong robustness of smooth variable structure estimation and the fault-tolerant capability of adaptive estimation. It compensates for attitude errors, mitigates the adverse impact of abnormal measurements, and yields lower attitude, velocity and position errors with fast convergence, which effectively improves the overall performance of SINS/GNSS integrated navigation under fault conditions.

**Figure 5:**
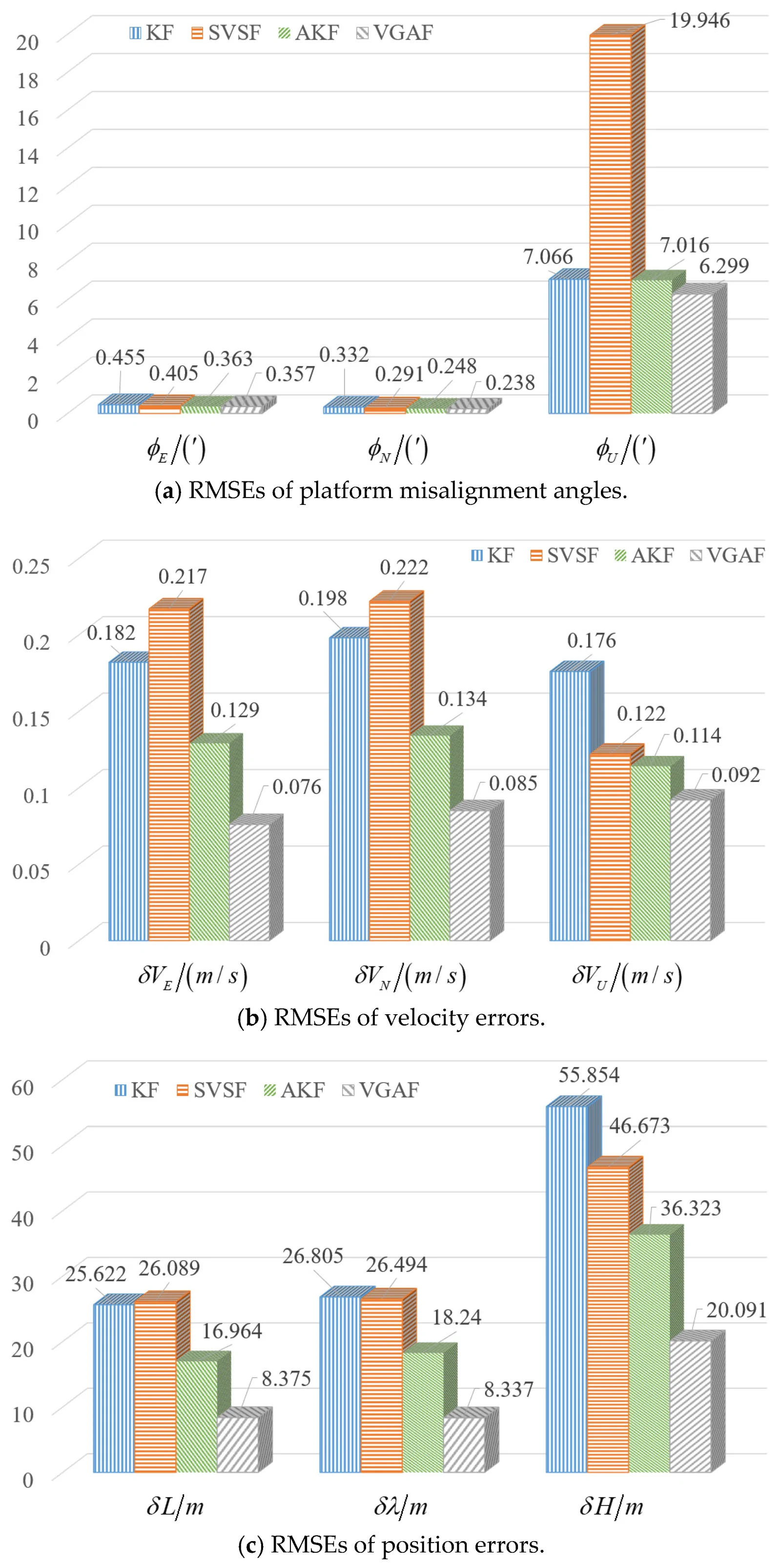
RMSEs of navigation errors from the four algorithms.

Root Mean Square Errors (RMSEs) are adopted as quantitative indicators to further evaluate the navigation performance of different methods. The RMSEs of integrated navigation platform misalignment angles, velocity errors, and position errors obtained from the above four algorithms are shown in Figure 5.

As can be seen from the statistical results in Figure 5, the estimation accuracy of Kalman filter degrades under abnormal measurement information. Constrained by its algorithmic principle, the smooth variable structure estimation also loses its robustness under the dual interference of attitude errors and measurement anomalies. The chi-square detection-based AKF can suppress partial abnormal measurement errors and obtain better estimation accuracy, but its performance degrades for minor and gradual change faults. However, the variable gain robust estimation algorithm proposed in this paper can monitor the quality of measurement information in real time based on the smooth bounded layer, and then adjust and reconstruct the filter gain. It has good robustness and stability against abnormal measurements, and achieves optimal comprehensive performance in terms of estimation accuracy, robustness and stability.

The computational performance of the algorithms is tested on a MATLAB (v2016) simulation platform equipped with an Intel (R) Core (TM) i5-8250U CPU @ 1.60 GHz, and the filter period of integrated navigation is set to 1 s. The average single-step filtering times of KF, SVSF, AKF and the proposed VGAF are 11.795 ms, 12.4751 ms, 12.4396 ms and 13.6672 ms, with corresponding average memory consumption of 7.6289 MB, 9.4063 MB, 9.7658 MB and 10.7813 MB, respectively. The computation times of the four algorithms are on the same order of magnitude, and all single-step runtime values are far shorter than the 1 s filter period, which can meet the real-time application requirements for UAV navigation. Compared with the basic KF, SVSF introduces the calculation logic of the smooth bounded layer, resulting in increases in both single-step runtime and memory overhead. AKF introduces a parameter adaptive adjustment mechanism to improve filtering adaptability, accompanied by a slight rise in computational load and memory consumption, and its computational overhead is roughly equivalent to that of SVSF. On the basis of SVSF, the proposed VGAF algorithm further incorporates the dynamic estimation module for measurement errors. Consequently, its computational overhead and memory usage rise moderately without a sharp surge in computing resource consumption, which demonstrates the feasibility of VGAF for practical engineering deployment in UAV navigation.

### 5.2. Ablation Simulations and Analysis

To clarify the individual contributions of the measurement-quality evaluation module, SVSF gain and adaptive Kalman gain in the VGAF algorithm, ablation simulations are carried out under the fault scenario given in Table 4. With identical noise parameters, fault injection schemes and fault-detection threshold, three ablation variants are constructed with clear functional definitions as follows.

(1) No-MQE: The measurement quality evaluation module is completely removed. No fault discrimination and mode switching are performed, and the standard Kalman filter runs throughout the whole simulation process.
(2) No-SVSF: The measurement quality evaluation and threshold-driven switching logic are retained. When measurements are abnormal, only the adaptive reconstructed robust gain is adopted for all states, and the SVSF gain module is disabled.
(3) No-AKF: The measurement quality evaluation and threshold-driven switching logic are retained. When measurements are abnormal, all states are updated merely by the SVSF gain, and the gain-reconstruction module for non-measurable states is turned off.

**Figure 6:**
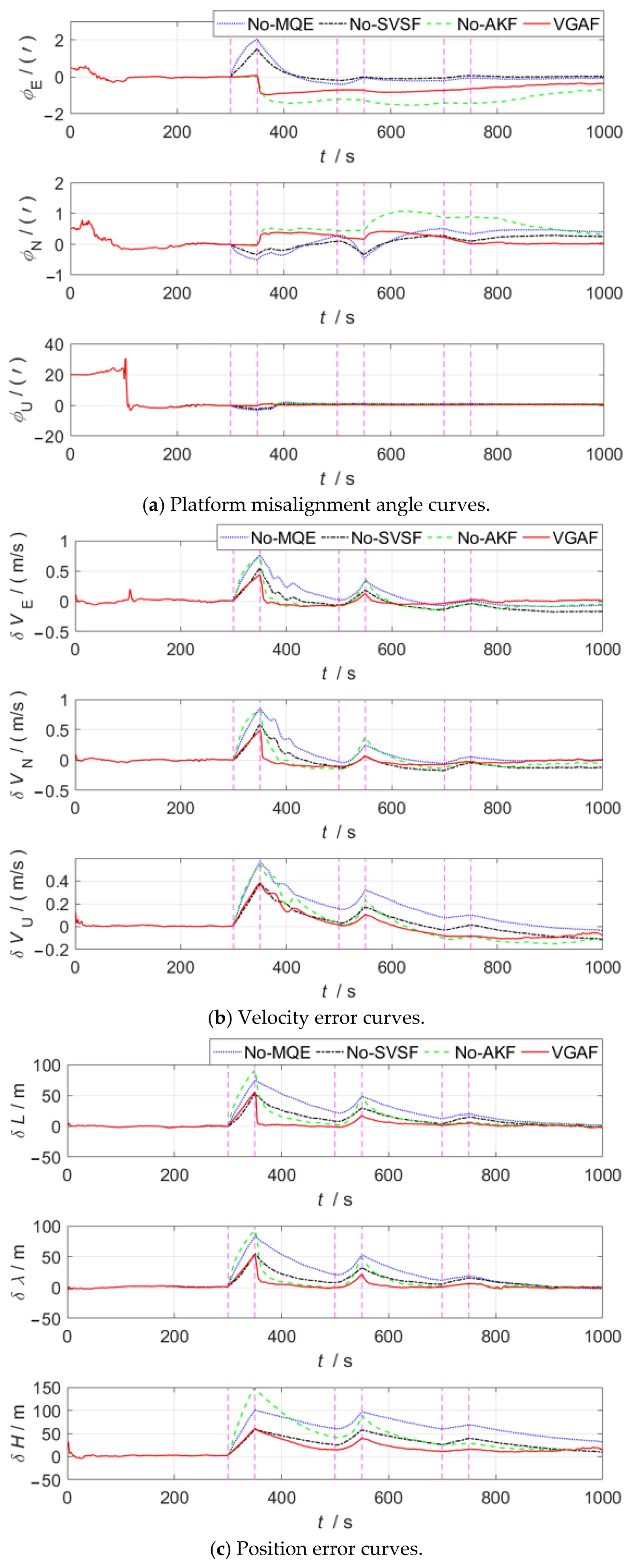
Error curves of the ablation experiments.

The platform misalignment angle curves, velocity and position error curves of different variants are presented in Figure 6.

The RMSEs of navigation errors obtained from the ablation experiments are listed in Table 6.

It can be observed that No-MQE yields the largest overall error, demonstrating that the measurement quality evaluation module serves as the fundamental component to suppress fault contamination and guarantee robust navigation.

Compared with No-AKF, No-SVSF achieves lower navigation errors, which illustrates the advantage of the adaptive Kalman gain in protecting attitude estimation and restraining position and velocity errors. It can be observed from Figure 6 that No-AKF converges faster than No-SVSF, reflecting that the smooth variable structure gain continuously constrains state uncertainty during faults and accelerates the attenuation of navigation errors.

The complete VGAF, integrating all modules, achieves superior navigation accuracy in all attitude, velocity and position dimensions.

### 5.3. Monte Carlo Statistical Simulations

**Figure 7:**
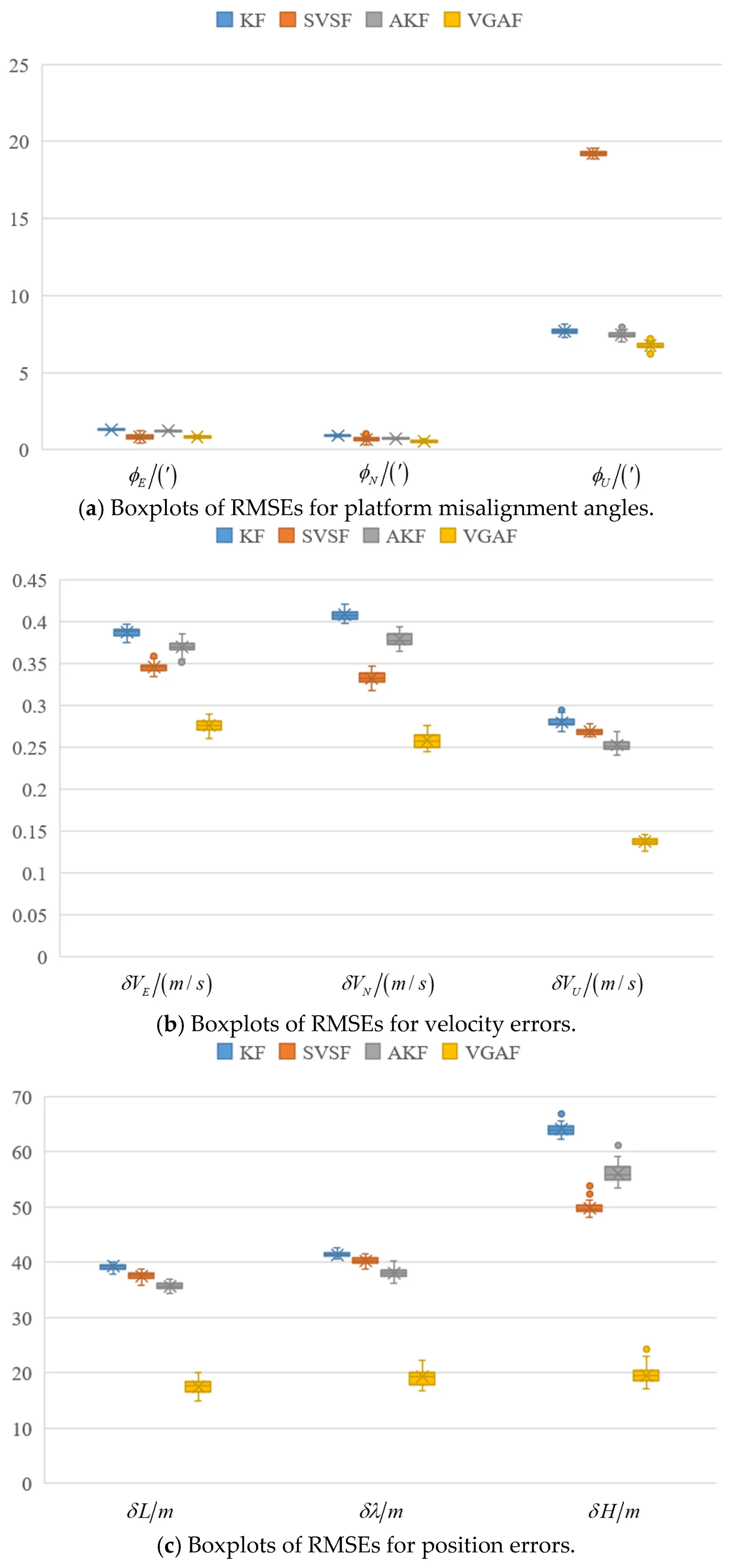
Boxplots of RMSEs for navigation errors obtained from Monte Carlo simulations.

To further verify the statistical reliability of the performance gains achieved by the proposed VGAF algorithm and test its robustness under degraded sensor conditions, Monte Carlo simulations are conducted with increased sensor noise. In this test, a new flight trajectory is adopted, where GNSS fault amplitudes and durations are randomized. A total of 100 independent Monte Carlo runs are executed to guarantee statistical significance. The inertial sensor precision and GNSS precision are degraded to 10-fold and 1.5-fold of their respective baseline values in Table 3, respectively, simulating the scenario of low-precision inertial devices and deteriorated GNSS observation quality. The boxplots of RMSEs for platform misalignment angles, velocity errors and position errors for KF, SVSF, AKF and the proposed VGAF are illustrated in Figure 7.

It can be observed from the Monte Carlo statistical results in Figure 7 that under the new trajectory with increased sensor noise and randomized fault amplitudes and durations, the proposed VGAF algorithm achieves smaller estimation errors and superior navigation performance compared with the other three algorithms.

In summary, the proposed variable gain robust estimation algorithm performs real-time measurement quality evaluation by using the width of the smooth bounded layer. When abnormal measurements occur, an equivalent weight matrix is constructed. Combined with smooth variable structure estimation and robust adaptive estimation, the algorithm independently adjusts each component of the filtering gain, suppresses error contamination caused by sensor noise and faulty measurements, and maintains favorable navigation accuracy. The statistical reliability of the performance gains achieved by the proposed method is therefore verified.

Based on the statistical RMSE results of 100 Monte Carlo runs, compared with KF, SVSF and AKF, the proposed VGAF improves the platform misalignment angle accuracy by more than 10%, velocity accuracy by more than 26%, and position accuracy by more than 56%, which supports the quantitative conclusion given in the Abstract.

### 5.4. Vehicle Experiments and Analysis

**Figure 8:**
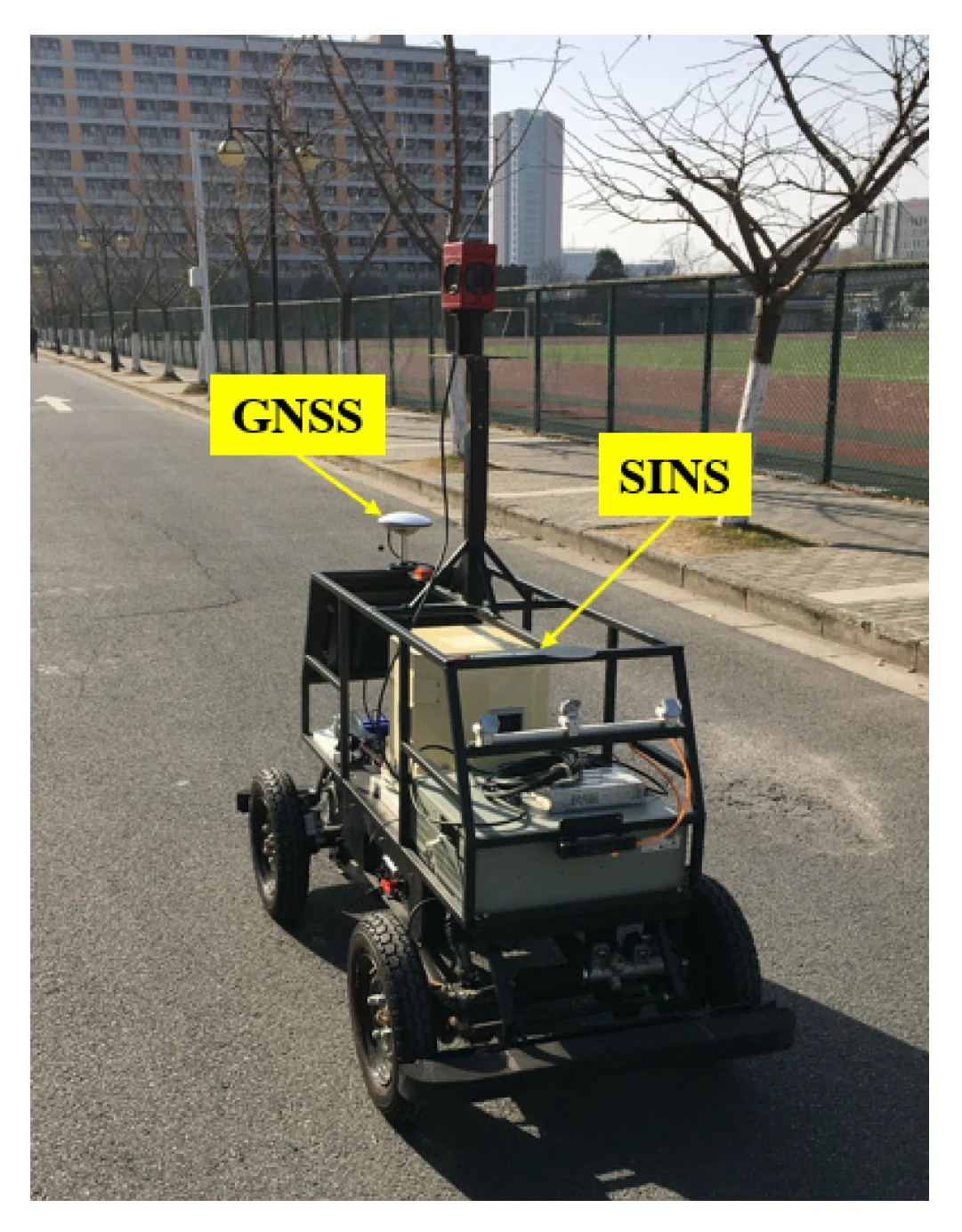
Vehicle-borne integrated navigation experimental platform.

To evaluate the performance and applicability of the proposed VGAF algorithm, a vehicle-borne integrated navigation field test is carried out under natural outdoor conditions in Nanjing, China. The test site is approximately at 118.790° E, 31.939° N. It is worth noting that the proposed VGAF algorithm is developed based on the general SINS/GNSS filtering framework and does not rely on specific carrier dynamic characteristics; hence, it can be transplanted from UAV airborne scenarios to land-vehicle test scenarios. Figure 8 shows the test vehicle fitted with a SINS and a GNSS receiver.

The parameters of the vehicle-borne SINS and GNSS are shown in Table 7.

**Figure 9:**
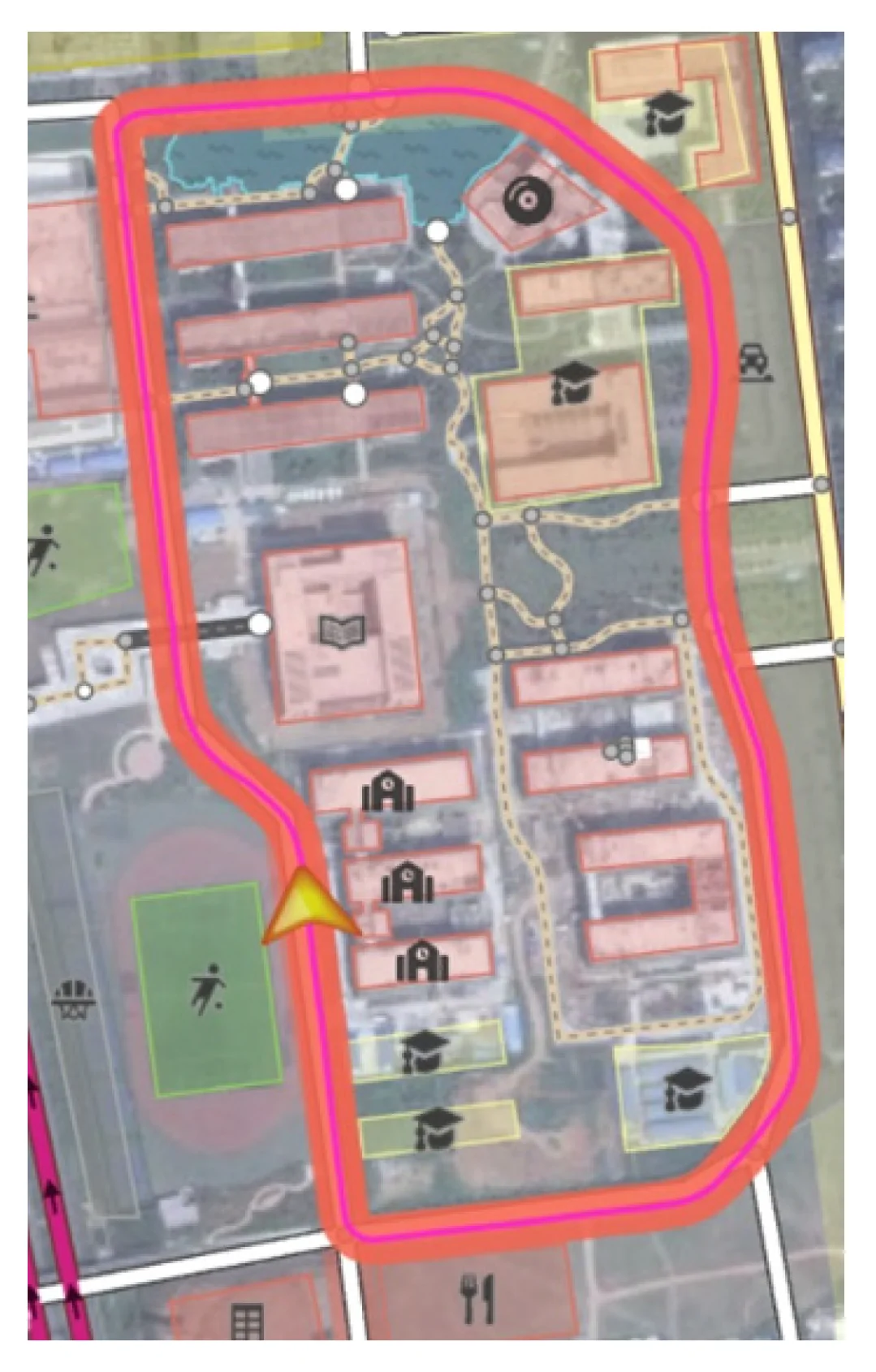
Vehicle trajectory.

The vehicle-borne integrated navigation system is equipped with RTK, which can provide high-precision navigation information and act as the reference to verify the accuracy of the integrated navigation algorithm. It should be noted that the RTK reference only provides position and velocity outputs without high-accuracy attitude information; hence, only horizontal velocity and position errors are evaluated in this vehicle-borne test. The field test was carried out on campus, and the vehicle trajectory is illustrated in Figure 9.

During the field test, satellite signals were contaminated by random errors arising from signal blockages and multipath effects induced by overpasses, trees and buildings along the test route. Such complex scenarios are adopted to verify the performance of the proposed VGAF algorithm.

**Figure 10:**
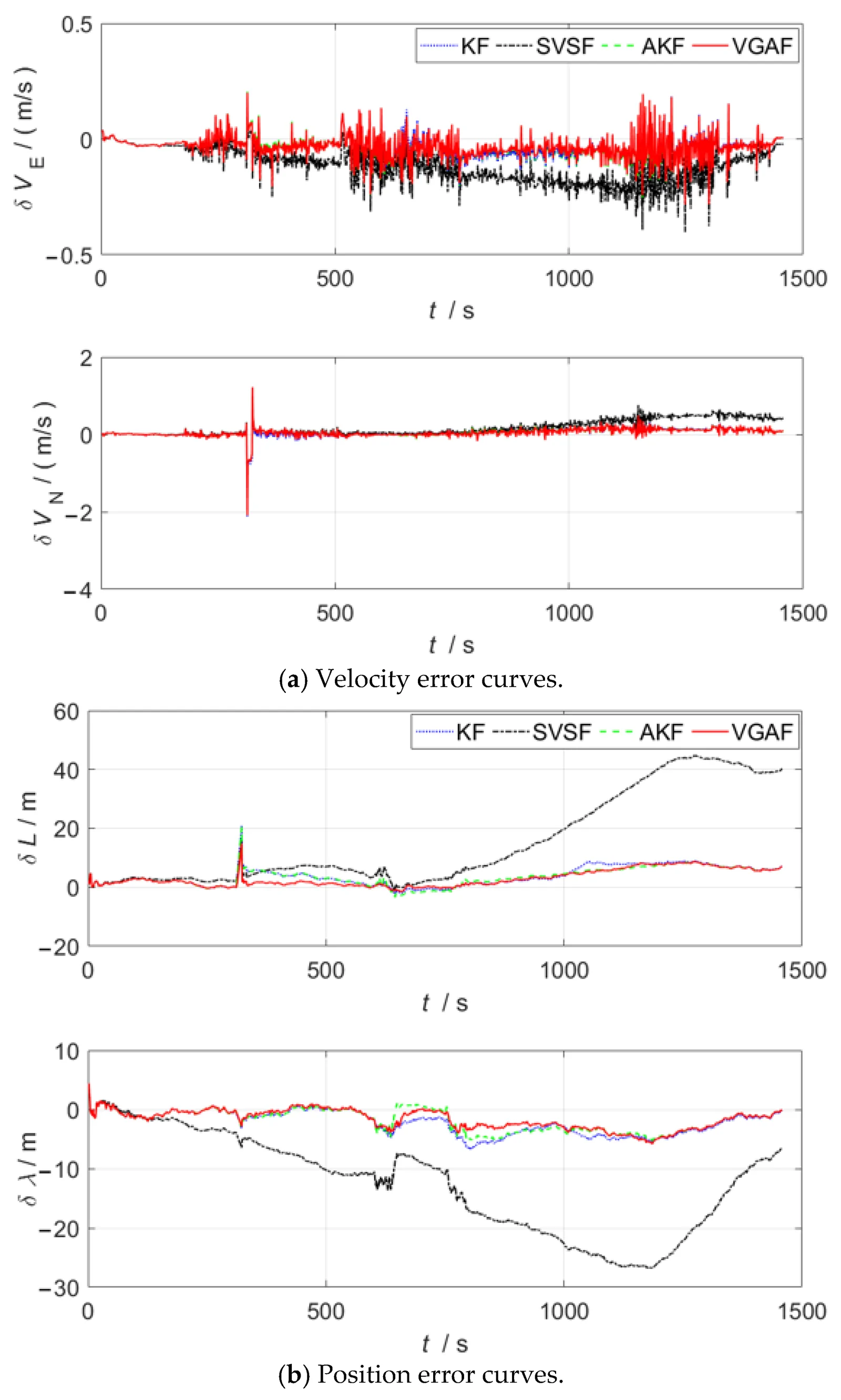
Vehicle experiment error curves of the four algorithms.

Taking the high-precision navigation information provided by RTK as the reference, the horizontal velocity error curves and horizontal position error curves of four algorithms, namely KF, SVSF, AKF and VGAF, are illustrated in Figure 10.

The RMSEs of navigation results obtained by the four algorithms in vehicle-borne experiments are listed in Table 8.

As can be observed from Figure 10 and Table 8, four algorithms yield different navigation performance under vehicle-borne tests with GNSS signal blockages.

Standard KF lacks fault-tolerant capability. Its estimation performance degrades significantly when GNSS observations are contaminated by outliers.

SVSF achieves the lowest accuracy. Restricted by its inherent principle, it cannot correct platform misalignment angles without corresponding measurements. Under satellite faults, the coupling of measurement anomalies and attitude errors leads to severe error fluctuations.

AKF utilizes the innovation chi-square test for fault detection. Its detection sensitivity is lower than that of the smooth bounded layer-based evaluation mechanism. Since the chi-square statistic, fault detection function and matrix adjustment factor are all scalars, AKF conducts fault judgment and adaptive adjustment for the entire measurement vector, and cannot identify abnormal components within multi-dimensional measurements.

The proposed VGAF achieves the minimum velocity RMSE and horizontal position RMSE. Benefiting from the smooth bounded layer driven measurement quality evaluation mechanism, VGAF combines SVSF gain for measurement-corresponding states and reconstructed robust gain for attitude-related states without direct measurements. It effectively suppresses error spikes during fault intervals and rapidly converges after signal recovery.

The field test verifies that VGAF possesses superior robustness and navigation precision for practical SINS/GNSS integrated navigation in complex outdoor driving scenarios.

## 6. Conclusions

To improve the state estimation performance and enhance the robustness of the airborne SINS/GNSS integrated navigation system, this paper studies a variable gain robust estimation algorithm based on measurement quality evaluation. Under abnormal measurement conditions, the feasibility of this scheme is verified, and its integrated navigation performance is analyzed. The verification results show that the variable gain robust estimation algorithm proposed in this paper effectively reduces estimation error, maintains high state estimation accuracy under complex and changeable satellite faults, and achieves significantly improved robustness. Based on the Monte Carlo experimental results, compared with the Kalman filter, the smooth variable structure filter and the innovation-based adaptive Kalman filter, the proposed variable gain robust estimation method improves the platform misalignment angle accuracy by more than 10%, the velocity accuracy by more than 26%, and the position accuracy by more than 56%. The proposed method exhibits favorable applicability for the SINS/GNSS integrated navigation system and possesses important reference value for practical engineering applications. Its effectiveness for land-vehicle scenarios has been validated by the vehicle-borne field test.

## Figures and Tables

**Table 1:** Detection performance indicators under different thresholds.

Thresholds	c=2	c=2.5	c=3	c=3.5
Fault detection rate	99.35%	99.35%	98.69%	98.69%
Missed detection rate	0.65%	0.65%	1.31%	1.31%
False alarm rate	4.96%	1.53%	0.35%	0.35%
Major step fault (300 s–350 s) detection delay	0 s	0 s	0 s	0 s
Gradual change fault (500 s–550 s) detection delay	1 s	1 s	1 s	1 s
Minor step fault (700 s–750 s) detection delay	0 s	0 s	1 s	1 s

**Table 2:** Theoretical comparison among KF, SVSF, AKF and the proposed VGAF.

Algorithms	Measurement Quality Evaluation Index	Gain Adjustment	State Partitioning Strategy	Switching Logic	Main Characteristics
KF	No quality evaluation.	No adaptive reconstruction.	No partition; uniform state treatment.	No switching mechanism.	Optimal under ideal Gaussian model; degrades with GNSS anomalies.
SVSF	No independent quality evaluation.	SVSF variable structure gain; only corrects measurement-corresponding states.	No explicit partition; updates only measurable states.	No threshold switch.	Robust for measurable states; fails for non-measurable states in SINS/GNSS.
AKF (NIS chi-square-based)	Chi-square NIS scalar test.	Global covariance scaling; single adjustment factor.	No explicit partition; full-vector processing.	χ^2^ threshold triggered tuning.	Works for large faults; insensitive to gradual/small-amplitude faults; global-only adjustment.
Proposed VGAF	Smooth bounded layer-based index (prior + posterior errors).	Dimension-wise reconstruction via equivalent weight matrix; mixed SVSF/AKF gain for different states.	Partition into measurable/non-measurable states.	Threshold-driven switch with saturation anti-jump.	High fault sensitivity; component-wise gain adjustment.

**Table 3:** Simulation parameters of SINS/GNSS.

Sensors	Measurement	Noise	Value
SINS	Gyro	Constant bias	0.01 deg/h
		Angular random walk	0.001 deg/sqrt(h)
	Accelerometer	Constant bias	100 μg
		Velocity random walk	10 μg/sqrt(Hz)
GNSS	Velocity	Error noise	0.1 m/s
	Position	Error noise	10 m, 10 m, 30 m

**Table 4:** GNSS fault information.

Time (s)	Fault Type	Fault Value
300–350	velocity major step fault	1 m/s
	position major step fault	100 m, 100 m, 300 m
500–550	velocity gradual change fault	0.02 m/s^2^
	position gradual change fault	2 m/s, 2 m/s, 6 m/s
700–750	velocity minor step fault	0.2 m/s
	position minor step fault	20 m, 20 m, 60 m

**Table 5:** Fault detection statistical indicators.

Indicators	Smooth Bounded Layer Detection	Chi-Square Detection
Fault detection rate	98.69%	77.78%
Missed detection rate	1.31%	22.22%
False alarm rate	0.35%	0.23%
Major step fault (300 s–350 s) detection delay	0 s	0 s
Gradual change fault (500 s–550 s) detection delay	1 s	7 s
Minor step fault (700 s–750 s) detection delay	1 s	-

**Table 6:** RMSEs of navigation errors from ablation experiments.

Errors	No-MQE	No-SVSF	No-AKF	VGAF
$\phi_{E}/{(}^{\prime })$	0.4547	0.3983	1.0240	0.3574
$\phi_{N}/{(}^{\prime })$	0.3322	0.2796	0.5757	0.2382
$\phi_{U}/{(}^{\prime })$	7.0659	7.0122	7.0212	6.2990
$\delta V_{E}/\left(m/s\right)$	0.1824	0.1138	0.1524	0.0761
$\delta V_{N}/\left(m/s\right)$	0.1975	0.1201	0.1754	0.0850
$\delta V_{U}/\left(m/s\right)$	0.1756	0.1477	0.1538	0.0921
δλ/m	25.6216	12.9703	18.6309	8.3745
δL/m	26.8052	14.1420	18.4311	8.3372
δH/m	55.8541	38.1603	47.5430	20.0908

**Table 7:** Vehicle experiment parameters of SINS/GNSS.

Sensors	Measurement	Noise	Value	Frequency
SINS	Gyro	Constant bias	0.015 deg/h	200 Hz
		Angular random walk	0.0007 deg/sqrt(h)	200 Hz
	Accelerometer	Constant bias	85 μg	200 Hz
		Velocity random walk	10 μg/sqrt(Hz)	200 Hz
GNSS	Velocity	Error noise	0.1 m/s	1 Hz
	Position	Error noise	10 m, 10 m, 30 m	1 Hz

**Table 8:** RMSEs of navigation errors in vehicle-borne experiments.

Errors	KF	SVSF	AKF	VGAF
$\delta V_{E}/\left(m/s\right)$	0.0615	0.1352	0.0605	0.0586
$\delta V_{N}/\left(m/s\right)$	0.1325	0.2700	0.1298	0.1282
δL/m	4.8701	21.7604	4.6093	4.1496
δλ/m	2.9443	14.9282	2.5918	2.3418

## Data Availability

The original contributions presented in this study are included in the article. Further inquiries can be directed to the corresponding author.
